# Management of Inferior vena cava injury in a resource limited setup: A rare case report

**DOI:** 10.1016/j.ijscr.2024.110685

**Published:** 2024-11-28

**Authors:** Nurhussen Mossa Ahmed, Belete Shikuro Aki, Dawit Argaw Demeke, Sitotaw Mossa Ahmed

**Affiliations:** a Yekatit 12 Hospital Medical College, department of surgery, Addis Ababa, Ethiopia; b ALERT comprehensive specialized Hospital, department of surgery, Addis Ababa, Ethiopia; c Jinka University, department of statistics, Jinka, Ethiopia

**Keywords:** Inferior vena cava injury, Venorrhaphy, Duodenal injury, Gallbladder injury

## Abstract

**Introduction and importance:**

Traumatic injuries of the inferior Vena Cava (IVC) are rare among traumatic abdominal injuries. It accounts for fewer than 5 % of penetrating injuries and 0.5 % of blunt trauma injuries. Inferior vena cava injury has high Prehospital (30 % -50 %) and in-hospital (20 % - 66 %) mortality rates. Preoperative diagnosis of IVC injury is extremely difficult. Ligation, primary suture repair (venorrhaphy) and patch cavaplasty are among the management options for Inferior vena cava injury. Inferior vena cava injuries are rare and information is scarce especially in resource limited setups.

**Case presentation:**

A 22 years old female presented with right upper quadrant abdominal stab injury of 30 min duration. She was hypotensive and there was 3 × 2 cm right upper quadrant stab wound with breach of parietal peritoneum. The intraoperative finding was gallbladder perforation .duodenum through-through injury and suprarenal inferior vena cava 3 cm vertical laceration. Cholecystectomy, duodenal repair and direct suture repair (venorrhaphy) of IVC done. Post operatively patient had smooth course and discharged on her 9th pod day.

**Clinical discussion:**

The most frequently injured segment of the IVC is the infra-renal IVC (39 %), then the retro-hepatic IVC (19 %), the supra-renal IVC (18 %), the para-renal IVC (17 %) and the supra-hepatic IVC (7 %). The suprahepatic IVC has the highest mortality rate (100 %), followed by mortality rates of the retro hepatic IVC (78 %), juxtarenal IVC (50 %), suprarenal IVC (33 %), and infrarenal IVC (33 %). Operative management includes ligation, primary suture repair (venorrhaphy) and patch cavaplasty using saphenous vein graft, autogenously peritoneo-fascial (APF) graft, synthetic graft such as Gore-Tex and Dacron.

**Conclusion:**

Traumatic injury of the inferior Vena Cava is rare, however the mortality rate is high. Adequate resuscitation and early hemorrhage control (operation) can save the lives of IVC injured patients. We present a case of successful repair of IVC injury by venorrhaphy (suture repair) in a resource limited setup.

## Highlights

• Traumatic injuries of the inferior Vena Cava (IVC) are rare but mortality is high.
• We report a rare case of suprarenal inferior vena cava injury wit gallbladder and duodenal trauma in 22 years old female.
• She was operated and cholecystectomy, duodenal repair and IVC repair-venorrhaphy was done and discharged with smooth course.
• We present a case of successful repair of IVC injury by venorrhaphy (suture repair) in a resource limited setup.

## 1 Introduction

Traumatic injuries of the inferior Vena Cava (IVC) are rare among traumatic abdominal injuries, because of its retroperitoneal position and the protection by several intra-abdominal structures. It accounts for fewer than 5 % of penetrating and 0.5 % of blunt abdominal trauma injuries. The mortality rates for the IVC injuries are still high, with prehospital and in-hospital mortality rates of 30 % to 50 % and 20 % to 66 %, respectively. It can be due to inadequate or delayed fluid resuscitation, location of the injury, and difficulty of diagnosis and technical problems in repair [[1], [2], [3], [4], [5]]. Successful management of IVC injuries lies in immediate control of hemorrhage and repairing the vein in accessible cases. Preoperative diagnosis of IVC injury is extremely difficult if possible at all, nearly all patients are operated on the basis of findings suggesting hemorrhagic shock or peritonitis [6,7]. Injured IVC can be managed with inferior vena Caval ligation, direct suturing(venorrhaphy), venoplasty, end to end anastomosis, endovascular stenting or graft interposition with autogenous or synthetic materials [6,8]. IVC injuries are rare and information is scarce [9].Most publications on traumatic IVC injuries from Africa have been published from South Africa and West Africa with no reported case as far as we know from East Africa and Ethiopia. In the present case we report a successfully treated supra -renal IVC injury with associated duodenal and gallbladder Injury from a knife stab wound in a resource limited setup.

This case is reported in accordance with SCARE criteria [10].

## 2 Case presentation

**Fig. 1:**
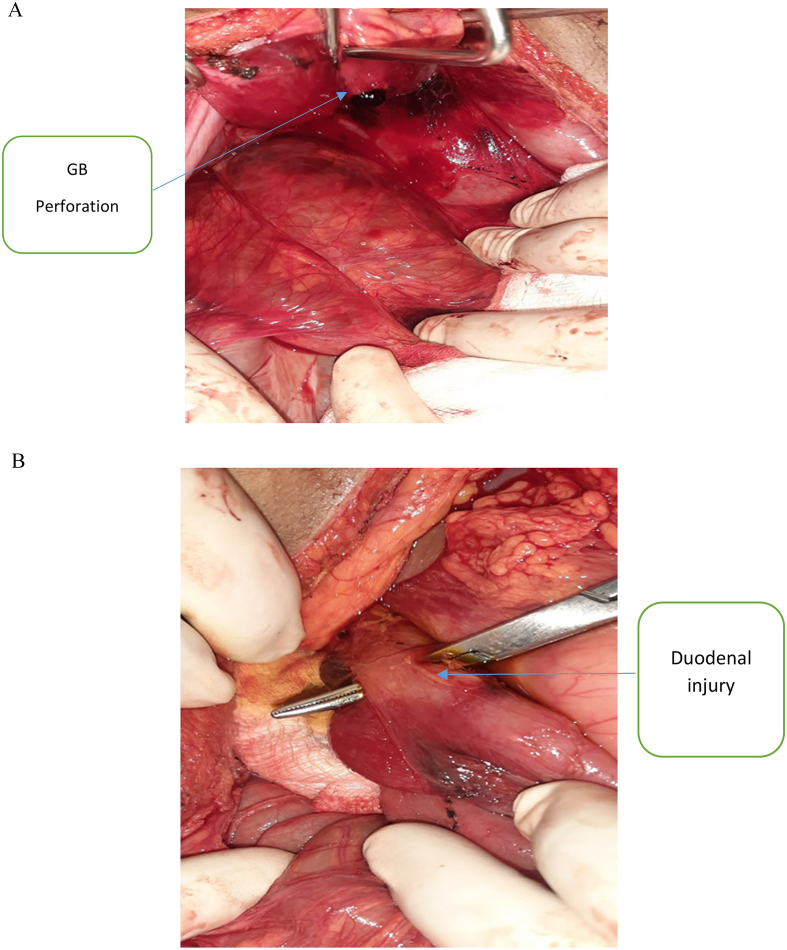
A. gallbladder body traumatic perforation oozing bile, B. second part of duodenal through - through penetration.

**Fig. 2:**
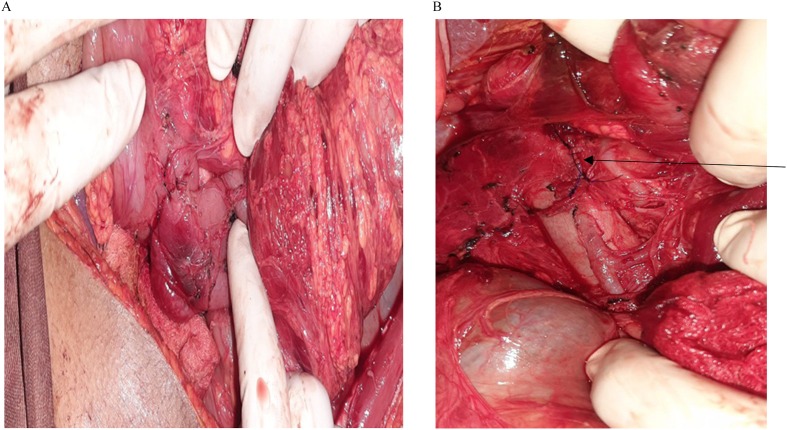
A. Repaired duodenum, B. Repaired inferior vena cava (black arrow).

**Fig. 3:**
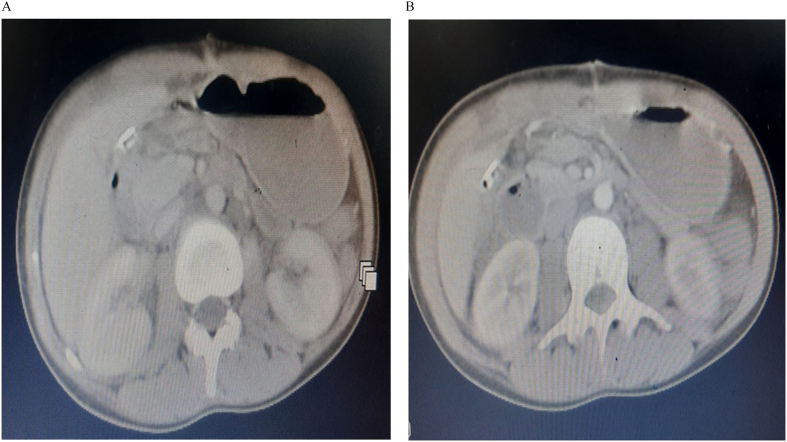
Post-operative abdominal CT scan of the patient.

A 22 year old female patient presented to emergency department after being stabbed on her anterior abdomen of 30 min duration. She complains pain and bleeding from right upper quadrant (RUQ) abdominal stab wound. Otherwise no vomiting, shortness of breath, loss of consciousness or trauma to other site. She had slightly pale conjunctiva, her pulse was 128 beat per minute and feeble, Blood pressure(BP) was 80/50, SPO2 of 94 % on room air, respiratory rate of 20 breaths/min. Glasgow coma scale(GCS) score of 15/15. The chest had clear and comparable air entry. The abdomen was full with moderate guarding and tenderness over RUQ area. There was bleeding through a 3x2cm stab wound on the right upper quadrant of the anterior abdominal wall about 2 cm above and lateral to the umbilicus with breach of the peritoneum. White blood cell count was 18,000, hemoglobin of 9 g/deciliter. Bed side abdominal ultrasound showed free fluid in pelvis, sub-hepatic area and rt. paracolic gutter. Despite intravenous crystalloid resuscitation and 2 unit of blood transfusion Blood pressure remains 88/60. With preoperative assessment of Hemorrhagic shock with hemoperitonium secondary to penetrating abdominal injury to rule out intra-abdominal vascular injury, patient rushed to operating theater for emergency laparotomy. Abdomen entered through vertical midline incision. There was about 400 ml hemoperitonium with retroperitoneal hematoma contained by Duodenum, right kidney and hepatic flexure part of transverse colon. Peritoneal blood sucked out, kockerisation of duodenum done. Intraoperative finding was: traumatic perforation of body of gall blader,1 × 1 cm through-through penetration of second part of duodenum (Fig. 1) and supra renal inferior vena cava 3 cm vertical laceration on the anteromedial side. Cholecystectomy done, duodenum repaired with interrupted vicril 3/0. IVC looped with glove rolled cuff distally and digital pressure control proximaly. IVC repaired with Prolene 4/0 in continuous fashion (Venorrhaphy) (Fig. 2).Nasogastric tube (NG) passed the duodenal repair. She received 2 unit preoperative and 2 unit intraoperative blood transfusion. Post operatively the vital signs were with in normal range with adequate urine output .The organ function tests were normal with serum creatinine of 0.8.She started sips through the NG tube and ambulation on 1st post-operative day (pod).NG tube removed on 4th pod after she tolerated oral fluid. Abdominal CT scan done on the 6th pod that revealed continuity of the IVC wall with no intraluminal thrombus (Fig. 3). She was anticoagulated with low molecular weight heparin (LMWH).The patient discharged on post-operative day 9.She was followed on outpatient surgical clinic for three month and the patient was in stable condition.

## 3 Discussion

Anatomically, the most frequently injured segment of the IVC is the infra-renal IVC (IRIVC) accounting 39 %, followed by the retro-hepatic IVC (RHIVC) (19 %), the supra-renal IVC (SRIVC) (18 %), the para-renal segment (PRIVC) (17 %) and the supra-hepatic segment (SHIVC) (7 %).While 30–50 % of patients with IVC injury will die before getting to the hospital, another 30–50 % of those who make it to the hospital will die despite surgery and efforts at resuscitation [[11], [12], [13]]. Predictors of High risk of mortality from traumatic IVC injury include a higher level or site of IVC injury, increasing number of associated injuries, hypotension on arrival, severe blood loss, increased transfusion requirements, a low GCS on arrival, and the type of procedure at surgery [11,12,14,15]. The two most important factors for postoperative survival with traumatic IVC rupture are (1) the hemodynamic condition of the patient on arrival and (2) the location of the Caval lesion [16].

The suprahepatic segment has the highest mortality rate(100 %), followed by the mortality rates of retro hepatic IVC (78 %), juxtarenal IVC (50 %), suprarenal IVC (33 %), and infrarenal IVC (33 %) [17]. More complex suprarenal injuries and injury that requires prosthetic repair are associated with higher mortality rates [16]. Active hemorrhage from the retro hepatic IVC is infrequently controlled; the radical hepatic mobilization to expose the retro hepatic injured IVC had an extremely high mortality rate and is not advisable unless active bleeding is present and cannot be contained by perihepatic packing. However, if packing fails to control the bleeding, direct repair of the injured site may be the only way to do so. Total hepatic vascular occlusion or an atriocaval shunt can be used to achieve a clear operative field during repair [16,[18], [19], [20], [21]].

In our case, the patient presented with hypotension, anemia requiring multiple transfusions, a GCS of 15/15, and suprarenal IVC injury and associated other organ injuries (gallbladder and duodenum). Her survival may have been due to her good GCS, since GCS is an independent risk for mortality in IVC injury [15] and early hemorrhage control, as delays in bleeding control in patients with significant abdominal injuries are associated with high mortality [22].

Operative management of IVC injuries include ligation, primary suture repair(venorrhaphy) and patch cavaplasty using saphenous vein graft, autogenous peritoneo-fascial (APF) graft, synthetic graft such as Gore-Tex and Dacron [13,23].

However, the surgical management for IVC injuries remains a matter of debate. Every case should be evaluated on its own merit and in all circumstances, the ultimate aim must be to stop the bleeding [5,23,24]. In the presence of a tamponade or contained hematoma a conservative approach to management has been advocated and found to have improved survival [14,24]. Van Rooyen et al. indicated that some IVC injuries are mostly missed, never operated without any consequence [24].

As primary repair may not always be possible, IVC ligation can be performed simply and quickly as part of damage control surgery in critically ill patients. In massive or smash injuries, temporary ligation in a damage control situation may be attempted. Suprarenal IVC should not be ligated as that would interrupt renal drainage and push the patient into renal failure. However, the incidence of compartment syndrome, pneumonia, deep venous thrombosis, and pulmonary embolism were significantly higher in patients who underwent IVC ligation [5,25].

Very high survival rates have been reported for suture repair (venorrhaphy) particularly for infra renal IVC injury [12,24]. venorrhaphy and cavaplasty using saphenous vein patch are relatively simple and can be done by the general surgeons [26].Our patient had 3 cm vertical laceration over suprarenal IVC and direct suturing technique was done with proline 4/0.Post-operative CT showed no narrowing or thrombosis of IVC. Sathiamurthy and Tan report an IVC injury where mobilization of the duodenum in a patient with a stab wound revealed a suprarenal IVC tear that had been tamponaded by a haematoma [27]. Similarly, in our patient a retroperitoneal clot had tamponaded the IVC tear and was noticed while mobilizing the duodenum (kockerisation).

Patients who survive IVC injuries tend to be long-term survivors regardless of the method of management, and complications are very uncommon [13].

Vena cava injuries caused by penetrating mechanism are invariably associated with other intraabdominal injuries that necessitate operative management such as perforation of other hollow viscus and injury to other solid organs [13,28]. Our patient sustained concomitant injuries of the duodenum and gallbladder that are managed with duodenal repair and cholecystectomy.

Long term anticoagulation is not an absolute indication unless in a more extensive injury, when a patch repair or graft is used depending on the hemodynamic stability of the patient [11]. Our patient was put on venous thromboprophylaxis for the first 5 days after surgery.

Some of the technical difficulty of the procedure in IVC injury management in resource limited setup like our Hospital are: - Glove cuff roll looping of the IVC may not totally stop the bleeding at injury site making repair difficult, Using assistant digit to compress the IVC proximal and distal to injury site against the vertebra is one option but when the assistant hand feels tired and loosens the compression, bleeding obscures the injury site and interrupts the repair multiple time.

## 4 Conclusion

Traumatic injury of the inferior Vena Cava is rare, however the mortality rate is high. Ligation, primary suture repair (venorrhaphy) and patch cavaplasty are among the management options for IVC injury. This case showed the lifesaving significance of early hemorrhage control in the management of IVC injury, as delays in hemorrhage control are associated with high mortality. Management of IVC injuries require multiple resources, including intensive care units, anesthetic care and blood products as well as team of trauma, general and vascular surgeons with vascular equipments. But some hospitals are far away from arranging such type of team and vascular sets. . We present a case of successful repair of IVC injury by venorrhaphy (suture repair) in a resource limited setup by general surgeons. We believe that this technique can be performed by general surgeons in resource limited setups (with no vascular sets and no vascular surgeons).

## Abbreviations

BP: blood pressure
IVC: Inferior vena cava
POD: Post-Operative Day
RUQ: right upper quadrant

## Author contribution

1. Nurhussen Mossa Ahmed: MD. General Surgery resident: Methodology, Conceptualized, wrote, reviewed and submitted the report. Operated on the patient and involved in follow up of the patient.
2. Belete Shikuro Aki: MD.General surgeon: involved in the management, review of the report and in the follow up of the patient
3. Dawit Argaw Demeke: MD, General surgery resident: involved in management of the patient and data acquisition
4. Sitotaw Mossa Ahmed: MSC, in Applied statistics: Involved in writing and reviewing the case report

## Patient consent

Written informed consent was obtained from the patient for publication of this case report and accompanying images. A copy of the written consent is available for review by the Editor-in-Chief of this journal on request.

## Ethical approval

Ethical approval was provided by the author's institution.

## Guarantor

Nurhussen Mossa Ahmed

Belete Shikuro Aki

## Source of funding

N/A

## Declaration of competing interest

All authors declare that they have no conflict of interest.
